# Supplementary material for: Crawling and Gliding: A Computational Model for Shape-Driven Cell Migration
Source: PLoS Comput Biol. 2015 Oct 21;11(10):e1004280. doi: 10.1371/journal.pcbi.1004280 (PMC4619082; doi:10.1371/journal.pcbi.1004280)
Supplement: S1 Code — (ZIP) [file pcbi.1004280.s012.zip › release/tst/doc/html/x11graph_8cpp.html]

Tissue Simulation Toolkit: x11graph.cpp File Reference


Tissue Simulation Toolkit
 0.1.4.1


- Main Page
- Namespaces
- Classes
- Files

- File List
- File Members

Macros |
Variables

x11graph.cpp File Reference

`#include <stdio.h>`  
`#include <unistd.h>`  
`#include <X11/Xlib.h>`  
`#include <X11/Xutil.h>`  
`#include <X11/keysym.h>`  
`#include <X11/cursorfont.h>`  
`#include <math.h>`  
`#include <malloc.h>`  
`#include <stdlib.h>`  
`#include <sys/types.h>`  
`#include <sys/stat.h>`  
`#include <fcntl.h>`  
`#include <cstring>`  
`#include <errno.h>`  
`#include <png.h>`  
`#include "sticky.h"`  
`#include "x11graph.h"`  
`#include "parameter.h"`  
`#include "output.h"`  
`#include <iostream>`  
`#include <string>`

Include dependency graph for x11graph.cpp:

|  |  |
| --- | --- |
| Macros | |
| #define | NOPVM |
|  | |
| #define | KEYBUFSIZE   10 |
|  | |
| #define | SWAP(a, b)   tmpswap = a; a = b; b = tmpswap; |
|  | |

|  |  |
| --- | --- |
| Variables | |
| Parameter | par |
|  | |
| int | errno |
|  | |

## Macro Definition Documentation

#define KEYBUFSIZE   10

Referenced by X11Graphics::GetXYCoo().

#define NOPVM

|  |  |  |  |
| --- | --- | --- | --- |
| #define SWAP | ( |  | a, |
|  |  |  | b |
|  | ) |  | tmpswap = a; a = b; b = tmpswap; |

Referenced by X11Graphics::Line().

## Variable Documentation

int errno

Referenced by CheckFile(), ReadLine(), and SearchToken().

Parameter par


---

Generated on Thu Aug 14 2014 22:04:01 for Tissue Simulation Toolkit by  

 1.8.6
